# A case of subcutaneous metastatic malignant melanoma of the left medial ankle: a case report and review of literature

**DOI:** 10.1186/s13256-024-04908-2

**Published:** 2024-12-31

**Authors:** Lauren Workman, Lauren Fang, Martina Blazevic, Joanna Chen, Richard Simman

**Affiliations:** 1 College of Medicine and Life Sciences, University of Toledo, 3000 Arlington Ave, Toledo, OH 43614 USA; 2 College of Medicine and Life Sciences, Division of Plastic and Reconstructive Surgery, University of Toledo, 3000 Arlington Ave, Toledo, OH 43614 USA; 3 Wound Care Program, Jobst Vascular Institute, ProMedica Health Network, 2109 Hughes Drive, Suite 400, Toledo, OH 43606 USA

**Keywords:** Malignant melanoma, Subcutaneous melanoma, Metastatic melanoma, Melanoma of unknown primary, Trauma, Surgical excision, Case report

## Abstract

**Background:**

Although rare, melanoma confined to the dermis or subcutaneous tissue without evidence of a primary cutaneous site should provoke consideration of melanoma of unknown primary. This diagnosis carries a favorable prognosis when compared with cutaneous metastatic melanoma. Several hypotheses have been proposed for how melanoma of unknown primary develops, two of which were considered in our patient case: (1) spontaneous regression of the primary tumor following metastasis or (2) the traumatic implantation of ectopic melanocytic cells in other tissues, such as the subcutaneous tissue. Although not a true example of melanoma of unknown primary, our case is still noteworthy as it represents a unique instance of melanoma presenting subcutaneously from trauma to a preexisting epidermal nevus.

**Case presentation:**

We present the case of a 66-year-old non-Hispanic Caucasian male who initially sought evaluation for a nontender lump of the left groin. Ultrasound-guided needle biopsy demonstrated stage III malignant melanoma. Upon further history taking, it was discovered that he had a nevus of the left medial ankle that was subjected to traumatic removal. He later developed a subcutaneous nodule at the same site. Positron emission tomography scan results supported the histopathologic findings which demonstrated invasive melanoma centered in the subcutaneous tissue without an epidermal component. Following left inguinal lymph node dissection, the patient received adjuvant immunotherapy and radiation to the left inguinal area. At 6 months following completion of therapy, metastases were identified in the lungs, vertebra, ribs, and liver. The patient is currently receiving immunotherapy with ipilimumab-nivolumab.

**Conclusion:**

As our patient did not have a readily apparent primary epidermal melanoma site at presentation, consideration was given as to whether this case may represent a melanoma of unknown primary, as originally defined by Das Gupta. This case does not meet the proposed criteria, however, as the patient reported a preexisting nevus in the area that was subjected to traumatic removal. Instead, we postulate that this trauma allowed for implantation of melanocytes into the subcutaneous tissue that later resulted in a malignant melanoma.

## Background

When a patient presents with malignant melanoma without evidence of an epidermal component, the differential diagnosis may include melanoma of unknown primary (MUP) which can present as palpable lymphadenopathy or as a solitary dermal lesion. As defined by Das Gupta, MUP involves presence of melanoma in the subcutaneous tissue, lymph nodes, or visceral organs without a primary site of the skin, eye, or mucosal membranes [2]. He also proposed multiple exclusion criteria involving surgical removal of the eye or orbit, surgical or traumatic manipulation of the skin, and a non-thorough examination [2]. We present a unique case of metastatic melanoma found initially in the subcutaneous tissue and left inguinal lymph nodes without an epidermal component that does not fit the criteria for melanoma of unknown primary owing to a history of trauma to a nevus in the area in which the subcutaneous nodule later developed.

### Case description

We present a unique case of a 66-year-old non-Hispanic Caucasian male with a metastatic melanoma, which developed following traumatic implantation of a preexisting, overlying epidermal nevus into the subcutaneous tissue.

On initial presentation, our 66-year-old male patient sought evaluation for a 3-month history of a self-palpable, slowly enlarging, nontender lump in his left groin. The patient reported intermittent dull pain at the site; however, denied any systemic symptoms, such as fevers, night sweats, weight loss, abdominal pain, nausea/vomiting, shortness of breath, or weakness. His medical history was significant for hypertension, dyslipidemia, and obesity with a body mass index of 32 kg m^−2^. Ultrasound-guided needle biopsy of the left groin lump demonstrated a stage III malignant melanoma. On immunohistochemistry, the mass demonstrated diffuse and strong staining of the tumor cells with S-100, SOX10, and MART-1, supporting the diagnosis.

**Figure. 1:**
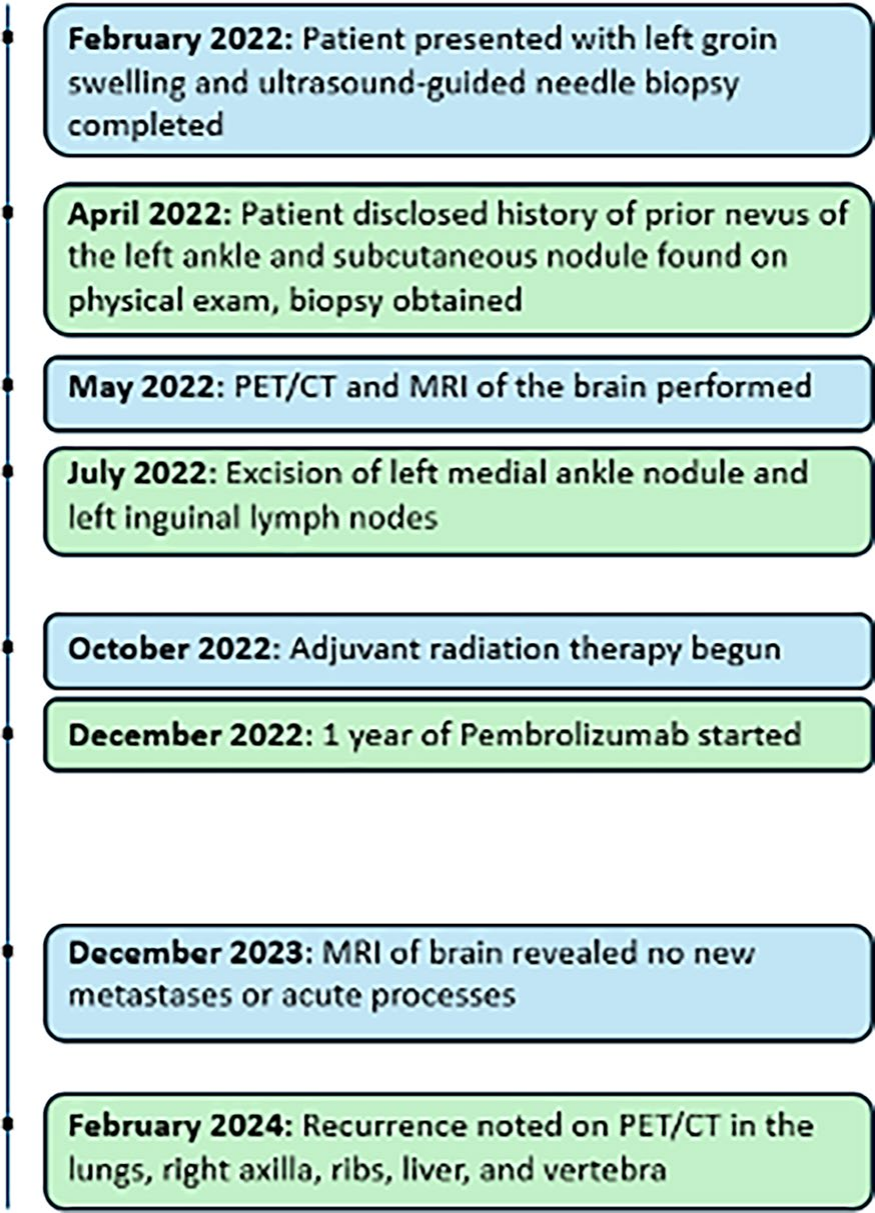
Timeline of care

Upon further history taking and investigation, the patient reported that 2 years prior to initial presentation, he had accidentally scraped off a “mole” on the medial aspect of his left ankle, that had been present since childhood, with his toenail. Although the mole disappeared when the skin healed, the patient noted the persistence of a deep, rock-hard nodule that was not present prior to the left medial ankle trauma. It was postulated that a prior nevus at the left medial ankle had been traumatized into the subcutaneous tissue and likely resulted in cutaneous metastatic melanoma presenting as a subcutaneous nodule with left inguinal lymph node swelling. The patient’s timeline of care is summarized in Fig. 1.

### Diagnostic assessment

Metastatic workup with a position emission tomography (PET) scan confirmed a 1.4 × 0.9 cm nodule in the left medial ankle with a standardized uptake value (SUV) of 21 and a left inguinal lymph node with SUV of 84. Magnetic resonance imaging of the brain was negative, and there were no other obvious metastatic lesions. Biopsy of the ankle mass located at the site of trauma confirmed a nodular type malignant melanoma of 8.9 mm Breslow depth without an epidermal component, ulceration, or lymphovascular invasion.

**Figure. 2:**
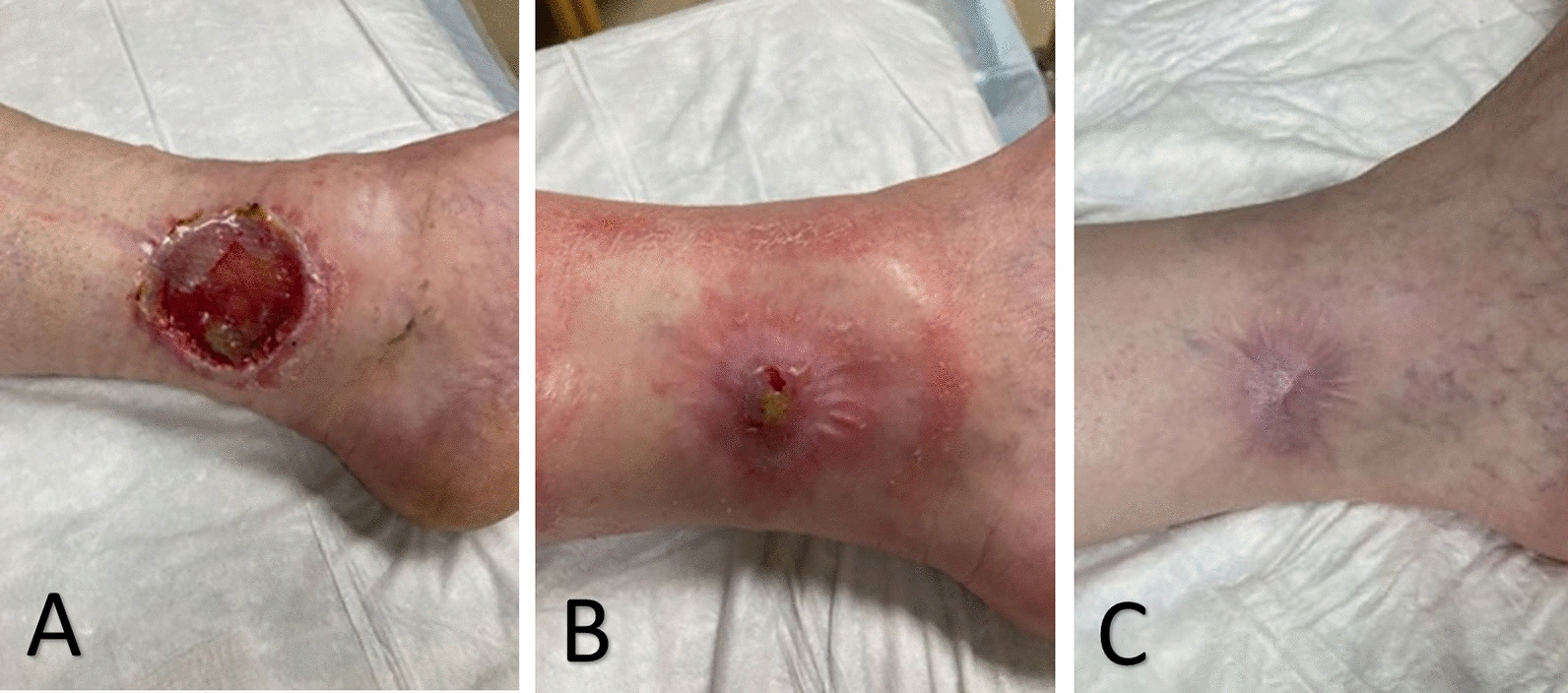
Healing progression following surgical removal of a subcutaneous melanoma **(A)** Left medial ankle wound 1 week following wide excision of subcutaneous nodule and application of Integra. **(B)** Healing left ankle wound 2 months postoperatively. **(C)** Healed left medial ankle wound 3 months postoperatively

The left medial ankle subcutaneous melanoma was surgically removed in the operating room with 3 cm wide excision and Integra application (Fig. 2A). At the same time, the left inguinal sentinel lymph node was excised, and biopsy was positive for melanoma. The left ankle wound healed 3 months after surgery (Fig. 2B, C). Using the American Joint Committee on Cancer (AJCC) 8th Edition, the final clinical staging was determined to be stage III (cT0, cN1b, cM0). The final pathologic staging was stage IIIC (pT4a, pN1b, cM0). Following lymph node dissection of the groin, the patient was treated with pembrolizumab every 3 weeks for 18 cycles and adjuvant radiation to the left inguinal area. Approximately 2 months following completion of immunotherapy, he was found to have multiple metastases to the lungs, vertebra, sternum, ribs, and right axillary lymph nodes that were fluorodeoxyglucose (FDG)-avid on PET/computed tomography (CT). Subsequent biopsy of a right axillary lymph node was positive for malignant melanoma. As such, he started immunotherapy again, this time with ipilimumab-nivolumab.

## Discussion and conclusion

### Excluding the diagnosis of MUP

Without an apparent epidermal component to the primary melanoma site, consideration was given to whether this case could represent a melanoma of unknown primary or cutaneous metastatic melanoma. MUP can present clinically as metastatic melanoma with palpable lymphadenopathy or as a solitary dermal lesion [1]. Approximately 2–9% of melanoma metastases appear without a known primary tumor [2–4]. There are several proposed hypotheses on the mechanism of MUP, which include: primary tumor regression after metastasis has already occurred, traumatic removal of the overlying epidermal component, and *de novo* melanomas arising within lymph nodes or in subcutaneous tissue. [5]

In 1963, Das Gupta originally defined MUP as melanoma found in the subcutaneous tissue, lymph nodes, or visceral organs without evidence of a primary site in the eye, mucosa, or skin [2, 6]. MUP presents most commonly in the lymph nodes (40–60% of all cases) followed by subcutaneous sites (30% of cases) [2]. Das Gupta also proposed exclusion criteria for MUP, which include: (1) evidence of previous orbital exenteration or enucleation; (2) evidence of previous skin excision, electrodessication, cauterization, or other surgical manipulation of a mole, freckle, birthmark, paronychia, or skin blemish; (3) evidence of metastatic melanoma in a draining lymph node with a scar in the area of skin supplying that lymph node basin; and (4) lack of thorough physical examination, including the absence of an ophthalmologic, anal, and genital exam [2, 6]. Despite these existing criteria, a recent systematic review of MUP conducted by Kamposioras *et al*. reported that only 16% of peer-reviewed articles and abstracts used the original Das Gupta criteria for the characterization of MUP. [2, 7]

Given our patient’s melanoma positive inguinal lymph node and subcutaneous nodule, MUP was considered as a differential diagnosis. However, the patient did not undergo an ophthalmologic, anal, and genital exam, which by Das Gupta’s criteria would exclude the diagnosis of MUP. Most importantly, because he had a likely primary lesion at the left ankle given his history of a previous nevus there that was removed via unintentional trauma, our patient’s presentation was most consistent with cutaneous metastatic melanoma.

Some authors have challenged Das Gupta’s exclusion criteria, calling into question the feasibility of extensive full-body physical examinations, especially with improved imaging techniques available [8, 9]. Tos *et al*. conducted a retrospective study of 103 patients diagnosed with metastatic MUP and concluded that only one possible (but not verified) primary tumor was disclosed by various specialist’s examinations [8]. Although PET/CT has been used for staging in patients with MUP, its role in identifying a possible primary site is not well studied [8, 9]. In our case, PET/CT was helpful in pinpointing the likely primary site.

### Prognostication and Survival of Stage-matched patients with MUP and MKP

As for the prognostication of MUP versus cutaneous metastatic melanoma, some authors have argued that MUP is associated with improved survival [2, 10]. Bae *et al*. conducted a meta-analysis of all studies with survival-to-date data and reported significantly improved overall survival for patients with stage III MUP compared with melanoma of known primary (MKP) patients (hazard ratio 0.83, 95% confidence interval 0.73–0.96, P = 0.01). According to Scott *et al*., MUP patients have better prognoses and improved overall survival compared with stage-matched patients with MKP, which supports the theory that an immune-mediated primary regression is an underlying mechanism that explains the development of MUP [2].

Other studies have found that the survival profile of patients with stage III MUP versus stage III MKP is not significantly different [4, 9, 11]. However, outcomes appear to change based on the number of metastatic lymph nodes involved in MUP [5, 12]. One study found that survival between MUP and MKP patients did not differ when only one lymph node was involved. However, in the presence of ≥ 2 metastatic lymph nodes, patients with MUP experienced significantly inferior outcomes compared with those with MKP [13]. Another study by van der Ploeg *et al*. concluded that more than three lymph node involvement was an independent prognostic factor of shorter disease-free and melanoma-specific survival in patients with MUP [14]. According to the eighth edition International Melanoma Database Kaplan–Meier melanoma-specific survival curves, the overall survival of stage IIIC melanoma is approximately 60% at 10 years since diagnosis. [15]

### Treatment of MUP and cutaneous metastatic melanoma

Patients classified as AJCC stage III, with or without a known primary cutaneous site, should be considered for surgery in addition to neoadjuvant and adjuvant therapies [4]. Those presenting with subcutaneous disease should receive wide local excision while patients with lymph node disease should be offered lymph node dissection [2, 4]. In cases of MUP, routine, long-term follow-up is crucial, as the occult primary lesion can manifest years after initial diagnosis [2].

### The role of trauma in the development of melanoma presenting subcutaneously

Although not a true example of MUP, our case is still noteworthy, as it represents a unique instance of melanoma presenting subcutaneously from trauma to a preexisting epidermal nevus. Prior studies have shown that mechanical pressure has various effects on skin and its cellular processes, which drive the development of certain cutaneous pathologies [16]. Melanocytes subjected to external forces can exhibit pathological changes that induce the development of melanoma [16]. Potential mechanisms include dysregulated cellular communication between melanocytes and other cells, subcellular structural instability and damage, altered modulation of melanoblast differentiation, and tumorigenesis influenced by extracellular matrix stiffness [17–20]. Mechanical pressure has also been shown to promote tissue proliferation via epidermal cell-cycle arrest and upregulation of the proto-oncogene c-fos. [21]

Tattooing is a mechanically traumatizing procedure involving the introduction of exogenous pigment into the dermis by puncturing the skin [22]. Several cases of melanoma arising in tattoos have been reported throughout literature, although the pathophysiology remains unclear [22–25]. Dermabrasion is another form of controlled mechanical trauma which involves “sanding” the skin using a rapidly rotating device. A combined histopatholgical and immunohistochemical study identified alterations in acquired melanocytic nevi after they underwent dermabrasion up to the superficial dermis [26]. At 4 weeks after the dermabrasion, atypical features including melanocytic atypia and pagetoid spread, which simulate melanoma in situ, were identified. Furthermore, these alterations persisted longer than those found after ultraviolet radiation, which is the single greatest risk factor for developing melanoma [26]. Surgical excision of benign melanocytic nevi, while frequently performed to assess for malignancy, can also induce trauma [27]. Occasionally, this trauma is associated with melanoma, as evidenced by the fact that neval remnants of precursor nevi are found in up to one third of melanomas [28]. Moreover, it is thought that surgical excision can modify the milieu of cytokines in postsurgical skin, such that an activated melanocytic phenotype is produced. [29]

Some authors have noted that trauma may be a cocarcinogenic factor for melanoma formation [23, 30, 31]. Kaskel *et al*. conducted a retrospective, questionnaire-based study and found that 9% of the 369 respondents who had excision of histopathologically confirmed primary melanoma considered an association between trauma and melanoma to be likely [23]. Of the 32 patients reporting trauma, 22 (70%) recalled a single traumatic event, such as a cut, blow, tattoo, or abrasion, prior to melanoma diagnosis. In total, ten patients (30%) reported persistent trauma from irritation secondary to tight shoes or clothes. A total of seven patients developed melanoma on an acquired or congenital nevus after mechanical injury. Nodular malignant melanoma was the diagnosis in 10 of the 32 trauma-associated cases and was the second most common subtype. The largest tumor thickness was 7 mm, and this lesion resulted from persistent irritation on the lower leg from tight socks. Kaskel *et al*. concluded that patients with acral melanoma were more likely to report a history of trauma compared with those with melanoma at other sites, although this was not statistically significant. Additionally, patients who reported trauma appeared to have higher tumor thickness than those who did not (1.1 mm versus 0.85 mm median thickness). Troyonova *et al*. also sought to analyze the role of mechanical injury on preexisting pigmented skin lesions via a case–control study of 156 patients with histologically proven and treated cutaneous malignant melanoma matched by age, sex, and geographic residence with a control group lacking any oncological diagnosis [31]. The odds ratio indicated that history of trauma on a preexisting pigmented skin lesion was associated with an elevated, but not statistically significant risk for melanoma formation [odds ratio (OR) = 1.35; 95% confidence interval (CI) 0.59–3.07; p > 0.05]. Repeated trauma was associated with an elevated, but not statistically significant risk for melanoma genesis (OR = 7.01; 95%CI 0.88–15.99; p > 0.05).

This manuscript highlights an unusual presentation of metastatic melanoma initially concerning for melanoma of unknown primary. The case has educational merit in that it sheds light on the possible role of trauma in the development of subcutaneous melanoma. It also reviews the nuances in diagnostic criteria of various melanoma subtypes.

## Data Availability

Not applicable.

## Abbreviations

MUP: Melanoma of unknown primary
PET: Position emission tomography
SUV: Standardized uptake value
AJCC: American joint committee on cancer
CT: Computed tomography
MKP: Melanoma of known primary
